# *Astragalus* polysacharin inhibits hepatocellular carcinoma-like phenotypes in a murine HCC model through repression of M2 polarization of tumour-associated macrophages

**DOI:** 10.1080/13880209.2021.1991384

**Published:** 2021-11-02

**Authors:** Chun Li, Xin-You Pan, Mingyun Ma, Jun Zhao, Fengda Zhao, Ya-Ping Lv

**Affiliations:** a Department of Doppler Ultrasonic Medicine, The First Affiliated Hospital of Nanchang University, Nanchang, Jiangxi, China; b Combined Department of Traditional Chinese Medicine and West Medicine, The First Affiliated Hospital of Nanchang University, Nanchang, Jiangxi, China; c Department of Rheumatism Immunology, The First Affiliated Hospital of Nanchang University, Nanchang, Jiangxi, China

**Keywords:** Invasion, migration, proliferation, tumour growth

## Abstract

**Context:**

*Astragalus* polysaccharin (APS), an extract of *Astragalus propinquus* Schischk, exerts antitumor effects in hepatocellular carcinoma (HCC).

**Objective:**

This study investigated the mechanism of action of APS in HCC.

**Materials and methods:**

Tumour-associated macrophages (TAMs) were treated with APS (0, 8, 16 mg/mL) for 24 h. APS (16 mg/mL)-treated TAMs were co-cultured with MHCC97H/Huh7 cells for 24 h. Finally, BALB/c nude mice were divided into PBS, APS (50 mg/kg), APS (100 mg/kg), APS (200 mg/kg) groups: mice were inoculated with Huh7 cells to construct tumour xenograft model, followed by administration of APS (50, 100, 200 mg/kg) or PBS daily for 30 days. Cell proliferation, migration, invasion, tumour growth, macrophage markers and proportions were measured.

**Results:**

APS 16 mg/mL treatment enhanced the expression of M1 macrophage markers (iNOS, IL-1β and TNF-α) and M1 macrophage proportions, while reducing the expression of M2 macrophage markers (IL-10, Arg-1) and M2 macrophage proportions in TAMs. Moreover, the APS-mediated M1 phenotype of TAMs significantly repressed cell proliferation, migration and invasion of MHCC97H and Huh7 cells. Moreover, APS (50, 100, 200 mg/kg) enhanced M1 macrophage proportions and reduced M2 macrophage proportions in the tumour tissues, and thus inhibited tumour growth of HCC.

**Discussion and conclusions:**

APS inhibits HCC-like phenotypes in a murine HCC model through repression of M2 polarization of TAMs. This work provides a novel theoretical basis for the application of APS in the clinical treatment of HCC.

## Introduction

Primary liver cancer is a highly malignant tumour and ranks second in malignant tumour deaths (El Jabbour et al. 2019). Hepatocellular carcinoma (HCC) accounts for approximately 90% of primary liver cancers (Ringelhan et al. 2018). The prevention and treatment of HCC have become a major challenge for humans. Currently, the anti-HCC therapies include liver transplantation, liver resection, radiotherapy, systemic chemotherapy, interventional therapy, molecular targeted therapy and immunotherapy (Grandhi et al. 2016). However, the prognosis of HCC is relatively poor, and the high recurrence and metastasis rate of HCC after surgery is still one of the clinical problems. In China, clinicians widely use traditional Chinese medicine to treat HCC. Traditional Chinese medicine combined with modern medical treatment reduces the adverse effects of radiotherapy and chemotherapy drugs, improves cancer-related symptoms, thereby enhancing the quality of life of HCC patients (Xi and Minuk 2018).

*Astragalus propinquus* Schischk. is a commonly used traditional Chinese medicine for the clinical treatment of HCC. It is mainly composed of *Astragalus* polysacharin (APS) and astragaloside, which can enhance immune function (Jing et al. 2014). As the main active ingredient of *Astragalus propinquus*, APS has the advantages of broad-spectrum antitumor effect, safety and low toxicity (Zhang et al. 2019). Evidences show that APS exerts antitumor effects by regulating various signal transduction pathways, and thus induces tumour cell apoptosis and enhances the immune function (Chen et al. 2020; Guo et al. 2020). APS is used in combination with various chemotherapeutic drugs, which has the effect of reducing drug side effects and increasing antitumor effects (Zhai et al. 2019). A previous study has found that APS promotes apoptosis and represses the cell viability of HCC cells in a concentration-dependent manner (Huang et al. 2016). APS inhibits tumour growth of HCC in H22 cell-bearing mice by regulating antitumor activity-related immune-regulating properties (Lai et al. 2017). Compound extract of *Astragalus propinquus* and *Salvia miltiorrhiza* Bunge containing APS inhibits the progression of HCC through regulating miR-145/miR-21 mediated phosphorylation of Smad3 (Wu et al. 2019). However, the specific mechanism of action of APS in treating HCC is currently unclear.

The tumour microenvironment affects the occurrence and development of tumours, and the inflammatory microenvironment is an important part of the tumour microenvironment (Kim and Bae 2016). Many traditional Chinese medicines have significant intervention effects on the inflammatory microenvironment of tumours, and are an important means for the treatment of HCC (Wang et al. 2011; Ye et al. 2019). The activation of tumour-associated macrophages (TAMs), as an important part of the infiltrating white blood cells in tumour tissues, are closely related to the progression of HCC (Fu et al. 2019). The activated TAMs can be polarized into two types, namely classically (M1) and alternatively (M2) macrophages. M1 macrophages mainly participate in the inflammatory response in cooperation with various inflammatory factors. M2 macrophages are involved in tissue repair and remodelling, and are involved in tumour angiogenesis and promote tumour progression (Degroote et al. 2018). Previous studies have demonstrated that APS is involved in the regulation of macrophage activation (Yang et al. 2013; Li et al. 2019). Therefore, this study hypothesized that APS partakes in polarization of TAMs in HCC in order to inhibit HCC-like phenotypes including cell proliferation, migration and invasion.

## Materials and methods

### Cell culture and treatment

Human HCC cell lines MHCC97H and Huh7 were obtained from CCTCC (Wuhan, China). Human monocyte cell line THP-1 cells were purchased from ATCC (Manassas, VA, USA). These cells were cultured in Dulbecco’s modified eagle medium (DMEM, Invitrogen, San Diego, CA, USA) in the presence of 10% foetal bovine serum (FBS, Invitrogen) and 1% penicillin/streptomycin (Solarbio, Beijing, China). Cells were incubated in a constant temperature incubator at 37 °C and 5% CO_2_.

For macrophage differentiation, THP-1 monocytes were incubated with 25 ng/mL phorbol ester (PMA; Sigma-Aldrich, St. Louis, MO, USA) for 24 h, and then cultured in DMEM for another 24 h. The cell morphology of THP-1 monocytes and THP-1 macrophages was observed under an optical microscope (Olympus, Tokyo, Japan). THP-1 monocytes were suspended cells. PMA-induced THP-1 macrophages grew adherently, and the cell morphology was regular round or oval. The adherent THP-1 macrophages were collected for subsequent experiments. The expression of macrophage marker CD86 in the THP-1 monocytes and THP-1 macrophages was examined by flow cytometry. After that, THP-1 macrophages were incubated with the supernatant of Huh7 cells for induction of HCC-associated macrophages, TAMs. TAMs were incubated with different concentrations of APS (CAS number: 89250-26-0; purity > 98%; Aladdin, Shanghai, China) (0, 8, 16 mg/mL) for 24 h.

Co-culture assays were performed using 0.4 µm pored Transwell membrane inserts (Corning, NY, USA). THP-1 macrophages (M0), TAMs treated with PBS [TAM (0 mg/mL)] or TAMs treated with 16 mg/mL APS [TAM (16 mg/mL)] were seeded into the upper chamber. MHCC97H or Huh7 cells were seeded into the lower chamber. These cells were cultured in DMEM containing 10% FBS for 24 h.

### Gene expression

TRIzol reagent (Invitrogen) was utilized for total RNA extraction from cells and tissues. Total RNA was reverse transcribed into cDNA using the Gene Amp RNA PCR Kit (BioRad, Hercules, CA, USA). The relative expression of the gene was measured applying SYBR green PCR Master Mix (Applied Biosystems, Foster City, CA, United States). Quantitative real-time reverse transcription-PCR (qRT-PCR) was carried out in an ABI Prism 7700 real-time PCR machine (Applied Biosystems). The relative expression of genes was normalized to an internal gene (GAPDH) according to the 2^–ΔΔ^*^Ct^* formula.

### Flow cytometry

The proportions of M1 and M2 macrophages in TAMs were examined by flow cytometry as previously reported (Bamodu et al. 2019). In brief, TAMs suspension (100 μL) was incubated with 10 μL anti-human CD80-FITC (Thermo Fisher Scientific, Waltham, MA, USA) or anti-human CD206-APC (Thermo Fisher Scientific) antibodies at 4 °C for 1 h in the darkness. After that, TAMs were washed with PBS buffer, and then analyzed the proportions of M1 and M2 macrophages using BD FACScaliber flow cytometer (BD Biosciences, San Jose, CA, USA). The data were analyzed applying Cell Quest Software (BD Biosciences). THP-1 monocytes and PMA-induced THP-1 macrophages were stained with anti-human CD86-FITC (Thermo Fisher Scientific), and the expression of macrophage marker CD86 in these cells was examined by flow cytometry. CD80 and CD86 were used to mark M1 macrophages, and CD206 was used to mark M2 macrophages.

### Cell proliferation

CCK-8 assay was done to detect cell proliferation of MHCC97H and Huh7 cells using Cell Counting Kit-8 (Beyotime, Shanghai, China). MHCC97H and Huh7 cells were seeded into 96-well plates (2000 cells/100 μL) and then incubated with 10 µL of CCK-8 reagent at 37 °C for 2 h. After that, the absorbance of each well was detected at 450 nm wavelength using a microplate reader (Thermo Fisher Scientific).

### Cell migration and invasion

Cell migration and invasion of MHCC97H and Huh7 cells were examined utilizing a 24-well Transwell insert system (Corning). For migration assay, MHCC97H and Huh7 cells were seeded into FBS-free DMEM in the upper chamber. For invasion assay, MHCC97H and Huh7 cells were seeded into FBS-free DMEM in the matrigel-covered upper chamber. The lower chamber was supplemented with DMEM and 10% FBS. Cells were incubated at 37 °C with 5% CO_2_ for 24 h. The migrating and invading cells on the bottom surface of the chamber were stained with 0.5% crystal violet for 30 min. Five random visual fields were selected to observe cell migration and invasion under an optical microscope.

### Tumour-bearing mouse model

BALB/c nude male mice (age, 4–6 weeks; weight, 15–20 g; Beijing Vital River Laboratory Animal Technology Co., Ltd., China) were housed under SPF conditions with a 12 h light-dark cycle. Mice were randomly divided into 4 groups (*n* = 6): PBS, APS (50 mg/kg), APS (100 mg/kg) and APS (200 mg/kg). Mice were received a subcutaneous injection of Huh7 cells (2 × 10^6^ cells/100 μL) through the right forelimbs. Six days after inoculation, tumour-bearing mice were intraperitoneally injected with different doses of APS (50, 100, 200 mg/kg) once a day for 30 days. Tumour-bearing mice were intraperitoneally injected with PBS as control. During tumour growth, the tumour volume was measured every 5 days. Tumour volume was calculated as the following formula: tumour volume (cm^3^) = 1/2 × length × width^2^. Thirty days after inoculation, mice were euthanized by cervical dislocation, and the tumour tissues were rapidly excised. The tumour weight was assessed. After that, the tumour tissues were stored at −80 °C, or fixed with paraformaldehyde and embedded with paraffin for further use. All protocols were authorized by the Ethics Committee of The First Affiliated Hospital of Nanchang University.

### Immunohistochemical (IHC) staining

Paraffin sections (5 μm) of tumour tissues were received deparaffin and rehydration. The sections were treated with Target Retrieval Solution (Dako, CA, USA) and 3% H_2_O_2_. After that, the sections were incubated with the primary antibodies, CD68 (1:1000, Proteintech, Wuhan, China), CD86 (1:1000, Proteintech) or CD206 (1:10,000, Proteintech) at 4 °C overnight. The sections were then incubated HRP-IgG (1:500, Thermo Fisher Scientific) at room temperature for 30 min. Subsequently, the sections were stained with 3, 30-diaminobenzidine and haematoxylin. Six mice in each group and three tumour tissue sections of each mouse were used to evaluate the expression of CD68, CD86 and CD206 in the tumour tissues. The sections were observed under a microscope, and the positive cells were calculated utilizing Image J software (National Institutes of Health; Bethesda, Maryland, USA). CD68 was used to mark total macrophages, CD86 was used to mark M1 macrophages, and CD206 was used to mark M2 macrophages.

### Statistical analysis

Each assay was repeated for 3 times. All data were reported as mean ± standard deviation. GraphPad Prism 7 (GraphPad Software, Inc., La Jolla, CA, USA) was used for statistical analysis. Two-tailed Student’s *t*-test and two-way ANOVA were used to analyze the statistical difference between two groups or among multiple groups. *p* < 0.05 was considered statistically significant.

## Results

### APS promoted M1 polarization and inhibited M2 polarization in TAMs

**Figure 1.**
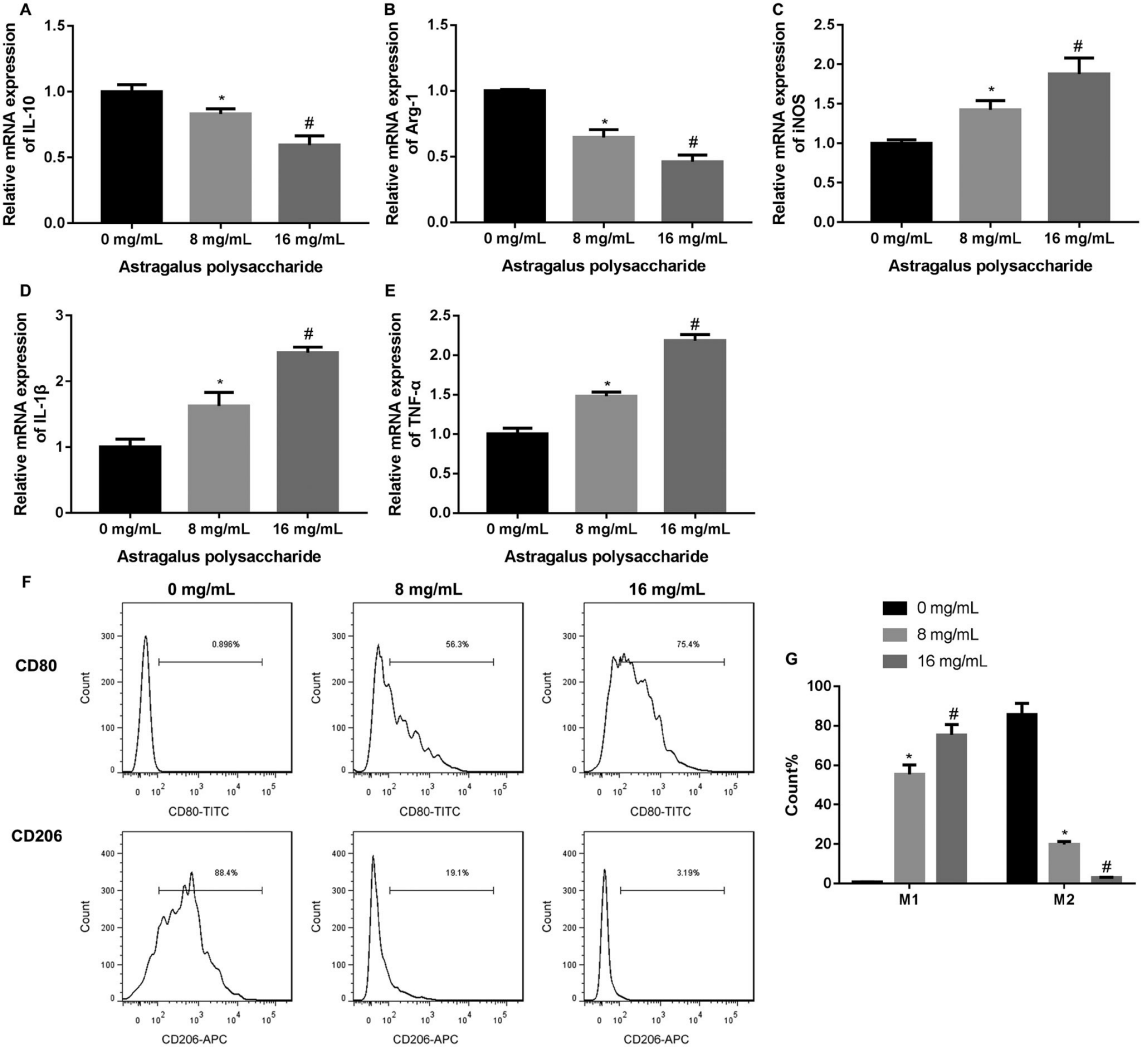
APS enhanced M1 macrophage proportions and reduced M2 macrophage proportions in TAMs. The qRT-PCR was performed to assess the expression of IL-10 (A), Arg-1 (B), iNOS (C), IL-1β (D) and TNF-α (E) in TAMs following the treatment of different concentrations of APS (0, 8, 16 mg/mL). (F–G) Flow cytometry was performed to estimate the proportions of M1 and M2 macrophages in TAMs following the treatment of different concentrations of APS (0, 8, 16 mg/mL). *p < 0.05 vs. 0 mg/mL; ^#^*p* < 0.05 vs. 8 mg/mL.

THP-1 monocytes differentiated into THP-1 macrophages induced by PMA. THP-1 monocytes were suspended cells. PMA-induced THP-1 macrophages grew adherently, and the cell morphology was regular round or oval (Supplementary Figure 1(A)). Then, flow cytometry was performed to assess the expression of macrophage marker CD86 in the THP-1 monocytes and THP-1 macrophages, showing that the expression of CD86 was significantly increased in THP-1 macrophages as compared with THP-1 monocytes (Supplementary Figure 1(B)). In order to detect the impact of APS on the polarization of TAMs, TAMs were treated with different concentrations of APS. The results of qRT-PCR revealed that APS treatment at 8 and 16 mg/mL severely reduced the expression of M2 macrophage markers IL-10 and Arg-1 in TAMs, although at different extents (Figure 1(A,B)). The expression of M1 macrophage markers iNOS, IL-1β and TNF-α was significantly increased in TAMs in the presence of 8 and 16 mg/mL APS. APS at 16 mg/mL exhibited a higher effect on the expression of macrophage markers than 8 mg/mL of APS (Figure 1(C–E)). Moreover, the proportions of M1 and M2 macrophages in TAMs were measured by flow cytometry, showing that both 8 and 16 mg/mL of APS enhanced the proportions of M1 macrophages and reduced the proportions of M2 macrophages in TAMs. APS at 16 mg/mL had a greater impact on the polarization of TAMs (Figure 1(F,G)). Thus, APS at 16 mg/mL was used to treat TAMs in the subsequent experiments. These data suggested that APS promoted M1 polarization and inhibited M2 polarization in TAMs.

### APS-treated TAMs inhibited cell proliferation, migration and invasion of HCC cells

**Figure 2.**
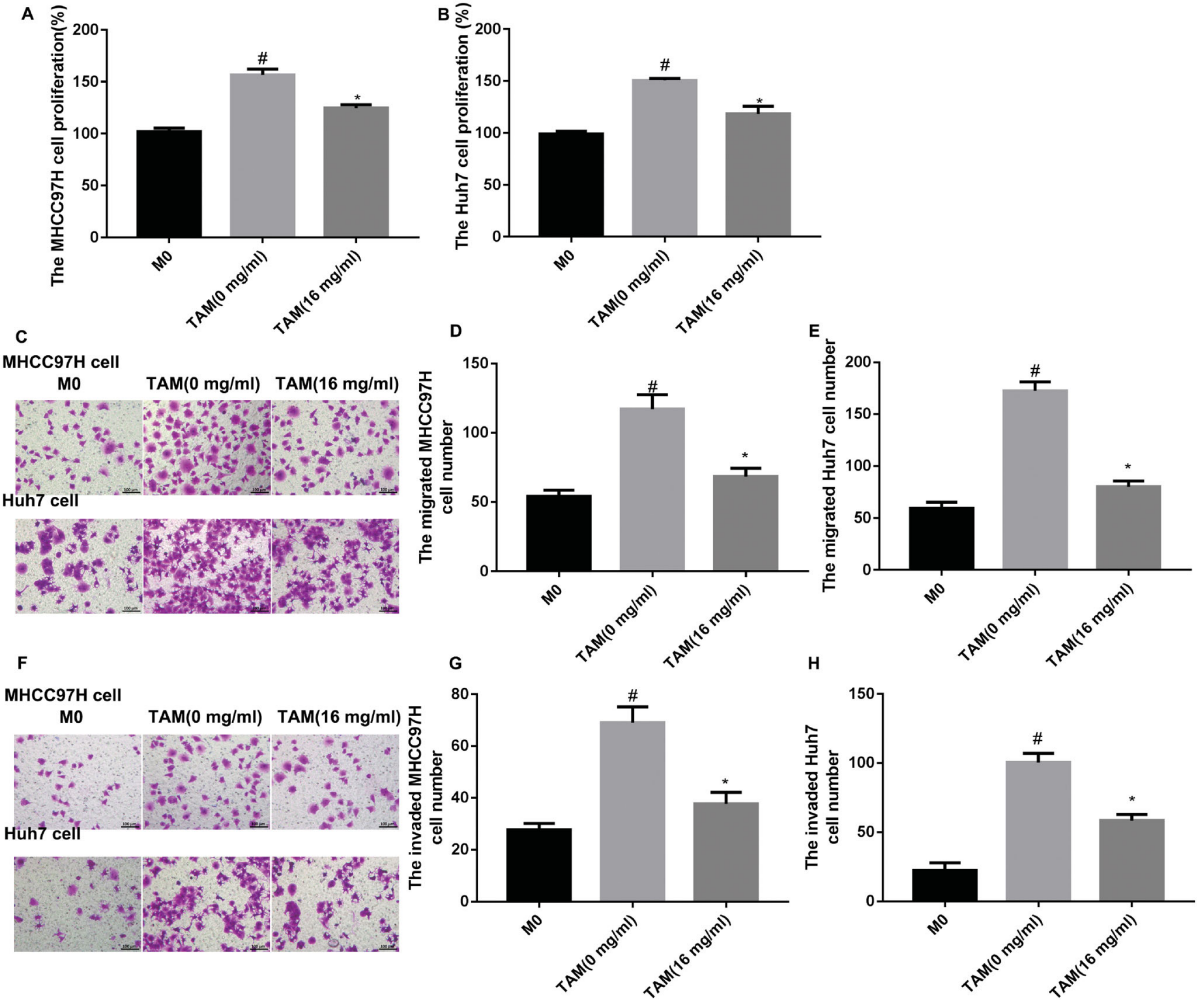
APS-treated TAMs promoted cell proliferation, migration and invasion of MHCC97H and Huh7 cells. TAMs were treated with PBS or 16 mg/mL APS, denoted as [TAM (0 mg/mL)] or [TAM (16 mg/mL)]. MHCC97H and Huh7 cells were co-cultured with THP-1 macrophages (M0), [TAM (0 mg/mL)] or [TAM (16 mg/mL)]. (A–B) CCK-8 assay was performed to examine cell proliferation of MHCC97H and Huh7 cells. (C–E) Cell migration of MHCC97H and Huh7 cells was assessed by Transwell migration assay. (F–H) Cell invasion of MHCC97H and Huh7 cells was detected by Transwell invasion assay. ^#^*p* < 0.05 vs. M0; *p < 0.05 vs. TAM (0 mg/mL).

Next, the impact of APS-treated TAMs on cell proliferation, migration and invasion of HCC cells was evaluated by CCK-8 and transwell assays. After co-cultured with TAMs, cell proliferation of MHCC97H and Huh7 cells was notably increased. APS-treated TAMs decreased cell proliferation of MHCC97H and Huh7 cells (Figure 2(A,B)). Further, transwell assays showed that cell migration of MHCC97H and Huh7 cells was enhanced following co-culture of TAMs, while migration ability of MHCC97H and Huh7 cells was severely decreased in the presence of APS-treated TAMs (Figure 2(C–E)). Moreover, TAMs promoted cell invasion of MHCC97H and Huh7 cells, whereas APS-treated TAMs repressed cell invasion of MHCC97H and Huh7 cells (Figure 2(F–H)). Thus, APS-treated TAMs inhibited cell proliferation, migration and invasion of HCC cells.

### APS inhibited tumour growth in HCC by inhibiting M2 polarization of TAMs

**Figure 3.**
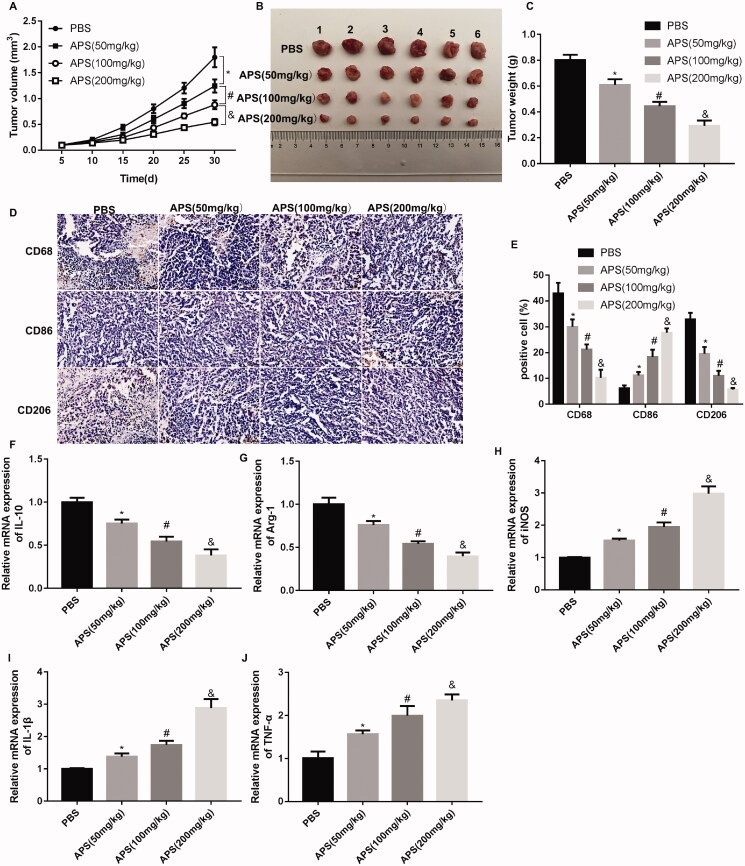
APS inhibited tumour growth in HCC by regulating polarization of TAMs. Tumour xenograft mouse model was constructed by inoculation of Huh7 cells, followed by administration of different concentration of APS (50, 100, 200 mg/kg) or PBS. The volume (A-B) and weight (C) of tumour were measured. (D-E) The expression of CD68 (macrophage marker), CD86 (M1 macrophage marker) and CD206 (M2 macrophage marker) in tumour tissues was assessed by IHC staining. The qRT-PCR was performed to assess the expression of IL-10 (F), Arg-1 (G), iNOS (H), IL-1β (I) and TNF-α (J) in tumour tissues. *p < 0.05 vs. PBS; ^#^*p* < 0.05 vs. APS (50 mg/kg); ^&^*p* < 0.05 vs. APS (100 mg/kg).

Finally, a tumour-bearing mouse model was constructed, followed by APS treatment. APS (50, 100, 200 mg/kg) administration effectively reduced the volume and weight of tumour in mice, especially 200 mg/kg APS (Figure 3(A–C)). Then, the expression of macrophage markers in tumour tissues was examined by IHC staining. As shown in Figure 3(D,E), APS (50, 100, 200 mg/kg) treatment reduced the levels of CD68-positive cells (macrophages) and CD206-positive cells (M2 macrophages), and enhanced the levels of CD86-positive cells (M1 macrophages). Furthermore, the results obtained from qRT-PCR showed that the expression of M2 macrophage markers IL-10 and Arg-1 was significantly decreased in tumour tissues of mice following different concentrations of APS treatment, although at different extent (Figure 3(F,G)). APS treatment led to an up-regulation of M1 macrophage markers iNOS, IL-1β and TNF-α in tumour tissues, especially APS at 200 mg/kg (Figure 3(H–J)). Taken together, APS inhibited tumour growth in HCC by inhibiting M2 polarization of TAMs.

## Discussion

APS has been used for the treatment of various diseases. For instance, APS protects human umbilical vein endothelial cells from oxidative damage triggered by H_2_O_2_ through promoting the expression of KLF2, thereby reducing the advancement of cardiovascular diseases (Li et al. 2019). APS also has antitumor effects in various cancers. APS induces the maturation of dendritic cells and effectively promotes the antigen-presenting capacity of dendritic cells and the activation of cytotoxic lymphocytes, and thus enhances the function of antitumor (Jing et al. 2014). The combination of APS and curcumin administration represses the tumour growth of HCC by promoting the maturation of tumour vessels and improving the morphological structure of tumour vessels (Tang et al. 2019). Compound extract of *Astragalus propinquus* and *Salvia miltiorrhiza* containing APS exerts antitumor effects on HCC by regulating miR-145/miR-21 axis, MAPK and TGF-β/Smad signalling pathways (Boye et al. 2015; Wu et al. 2019). APS inhibits cell proliferation and migration of Tregs by repressing the expression of FOXp3, which contributes to inhibit the immune suppressive effects of Tregs in the tumour microenvironment of HCC (Wu et al. 2018). However, little is known about whether APS can regulate polarization of TAMs to participate in the progression of HCC. In the present study, APS treatment enhanced the expression of M1 macrophage markers iNOS, IL-1β and TNF-α, and M1 macrophage proportions in TAMs. The expression of M2 macrophage markers IL-10 and Arg-1 and the proportions of M2 macrophage were decreased in TAMs following APS treatment. These data are consistent with the previous report, APS is able to induce much higher expression of M1 macrophage markers, including iNOS, IL-6, TNF-α, and CXCL10 in RAW264.7 cells (Wei et al. 2019). A previous study has confirmed that HCC-conditioned TAMs possess an M2-like phenotype, and highly express IL-10. The miR-98 represses IL-10 expression and inhibits M2 polarization of HCC-conditioned TAMs, thereby inhibiting the malignant phenotype of HCC cells (Li, Sun, Zhang, Li, Cui, et al. 2018). Moreover, miR-98 can inhibit cell migration and invasion of HepG2 cells by inhibiting IL-10 expression (Li, Sun, Zhang, Li, Zhou 2018). APS may affect polarization of TAMs through regulating miR-98/IL-10 axis. Thus, these data suggested that APS may regulate HCC development by regulating the polarization of TAMs.

TAMs are closely related to tumour growth and development. In the microenvironment of HCC, TAMs promote the sphere formation and tumour growth of cancer stem cells in HCC by secreting the proinflammatory cytokine IL-6 (Wan et al. 2014). Inhibition of NOTCH impedes TAMs differentiation, and promotes cell proliferation and the secretion of protumor cytokines of Kupffer cell-like TAMs by activating Wnt/β-catenin signalling pathway, thereby facilitating the progression of HCC and hepatic metastasis of colorectal cancer (Ye et al. 2019). M2 polarization of TAMs accelerates HCC progression by facilitating the ability of migration and epithelial-mesenchymal transition in SMMC-7721 and MHCC97-H cells (Yao et al. 2018). This study first revealed whether APS can regulate the polarization of TAMs to participate in HCC progression. APS treatment inhibited the M2 polarization of TAMs and promoted the M1 polarization of TAMs. APS-treated TAMs significantly repressed HCC-like phenotypes including cell proliferation, migration and invasion. In addition, administration of APS also reduced tumour growth of HCC in tumour-bearing mice. The expression of M1 macrophage markers iNOS, IL-1β, TNF-α and CD86 was increased, and the expression of M2 macrophage markers IL-10, Arg-1 and CD206 was reduced in the tumour tissues of tumour-bearing mice. Thus, these data confirmed that APS treatment inhibited tumour growth of HCC in mice by repressing M2 polarization of TAMs.

Previous studies have confirmed the inhibitory effect of APS on cell proliferation, migration, invasion of various cancers. For example, APS inhibits proliferation, migration and invasion while promotes apoptosis of human osteosarcoma cells, which attributes to promote miR-133a expression and then inactivate JNK signalling pathway (Chu et al. 2018). APS inhibits Wnt/β‑catenin signalling pathway to decrease cell proliferation, migration and invasion of breast cancer cells (Yang et al. 2020). Moreover, a previous study has confirmed that APS induces M1 polarization of macrophages via the Notch signalling pathway, and reduces the tumour growth in breast cancer-bearing mice (Wei et al. 2019). Thus, APS may affect the polarization of TAMs, cell proliferation, migration, invasion and tumour growth in HCC through miR-133a/JNK, Wnt/β-catenin and Notch signalling pathways. This study preliminarily determines the impact of APS on the polarization of TAMs and the progression of HCC, and its underlying mechanism is still unclear. This is the disadvantage of this article, and we will conduct in-depth research on this topic in future work.

## Conclusions

This work demonstrated that APS inhibits cell proliferation, migration, invasion and tumour growth in HCC by repressing M2 polarization of TAMs. Thus, this research provides a new theoretical basis for the application of APS in HCC treatment.

## Supplementary Material

Supplemental Material
