# Supplementary figures and images for: Astragalus polysacharin inhibits hepatocellular carcinoma-like phenotypes in a murine HCC model through repression of M2 polarization of tumour-associated macrophages
Source: Pharm Biol. 2021 Nov 2;59(1):1531–7. doi: 10.1080/13880209.2021.1991384 (PMC8567900; doi:10.1080/13880209.2021.1991384)

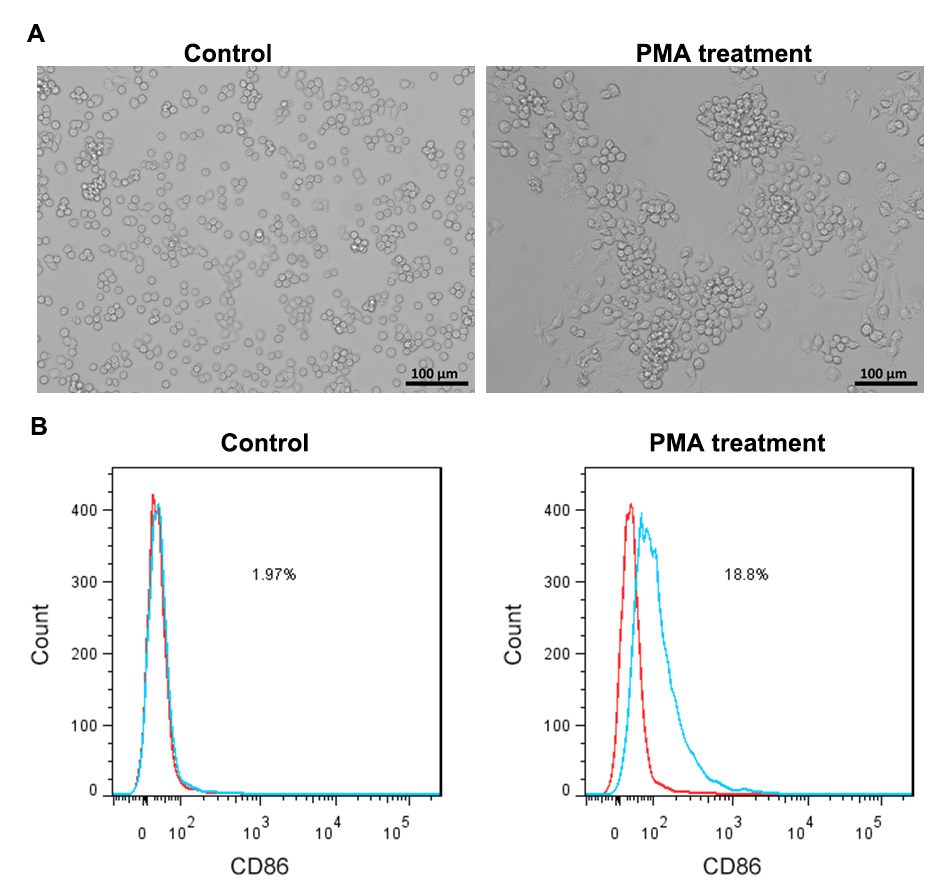

Supplement: Supplemental Material [file IPHB_A_1991384_SM6274.png]
